# Behavioral features in Prader-Willi syndrome (PWS): consensus paper from the International PWS Clinical Trial Consortium

**DOI:** 10.1186/s11689-021-09373-2

**Published:** 2021-06-21

**Authors:** Lauren Schwartz, Assumpta Caixàs, Anastasia Dimitropoulos, Elisabeth Dykens, Jessica Duis, Stewart Einfeld, Louise Gallagher, Anthony Holland, Lauren Rice, Elizabeth Roof, Parisa Salehi, Theresa Strong, Bonnie Taylor, Kate Woodcock

**Affiliations:** 1 Foundation for Prader-Willi Research, Walnut, CA USA; 2 Department of Rehabilitation Medicine, University of Washington School of Medicine, Seattle, WA USA; 3 Endocrinology and Nutrition Department, Parc Taulí University Hospital, Parc Taulí Research and Innovation Institute, Sabadell, Spain; 4 Medicine Department, Autonomous University of Barcelona, Sabadell, Spain; 5 Psychological Sciences, Case Western Reserve University, Cleveland, OH USA; 6 Vanderbilt Kennedy Center for Research on Human Development, Vanderbilt University, Nashville, TN USA; 7 Section of Genetics & Inherited Metabolic Diseases, Children’s Hospital Colorado, University of Colorado Anschutz Medical Campus, Aurora, CO USA; 8 Faculty of Medicine and Health, University of Sydney, Camperdown, NSW Australia; 9 Trinity College Dublin Trinity Translational Medicine Institute, St. James’s Hospital, Dublin, 8 Ireland; 10 Department of Psychiatry, Cambridge Intellectual and Developmental Disabilities Research Group, University of Cambridge, Cambridge, UK; 11 Brain and Mind Centre | Faculty of Health Sciences, The University of Sydney, Faculty of Medicine and Health, Camperdown, NSW Australia; 12 Department of Psychology and Human Development, Vanderbilt University, Nashville, TN USA; 13 Division of Endocrinology and Diabetes, Seattle Children’s, University of Washington, Seattle, WA USA; 14 Department of Genetics, University of Alabama at Birmingham, Birmingham, AL USA; 15 Department of Psychiatry and Behavioral Sciences, Albert Einstein College of Medicine, Bronx, NY USA; 16 Centre for Applied Psychology, School of Psychology, University of Birmingham, Edgbaston, Birmingham, UK

**Keywords:** Prader-Willi syndrome, Behavior, Hyperphagia, Temper outbursts, Anxiety, Obsessive–compulsive, Rigidity, Social cognition, Patient vignettes

## Abstract

Prader-Willi syndrome (PWS) is a rare neurodevelopmental genetic disorder associated with a characteristic behavioral phenotype that includes severe hyperphagia and a variety of other behavioral challenges such as temper outbursts and anxiety. These behaviors have a significant and dramatic impact on the daily functioning and quality of life for the person with PWS and their families. To date, effective therapies addressing these behavioral challenges have proven elusive, but several potential treatments are on the horizon. However, a limiting factor for treatment studies in PWS is the lack of consensus in the field regarding how to best define and measure the complex and interrelated behavioral features of this syndrome. The International PWS Clinical Trials Consortium (PWS-CTC, www.pwsctc.org) includes expert PWS scientists, clinicians, and patient advocacy organization representatives focused on facilitating clinical trials in this rare disease. To address the above gap in the field, members of the PWS-CTC “Behavior Outcomes Working Group” sought to develop a unified understanding of the key behavioral features in PWS and build a consensus regarding their definition and description. The primary focus of this paper is to present consensus definitions and descriptions of key phenotypic PWS behaviors including hyperphagia, temper outbursts, anxiety, obsessive–compulsive behaviors, rigidity, and social cognition deficits. Patient vignettes are provided to illustrate the interrelatedness and impact of these behaviors. We also review some available assessment tools as well as new instruments in development which may be useful in measuring these behavioral features in PWS.

## Background

Prader-Willi syndrome (PWS) is a rare genetic neurodevelopmental disorder that occurs in approximately 1 in 15,000 to 30,000 births, caused by a loss of paternally expressed imprinted genes on chromosome 15q11.2–q13 [1]. Genetic subtypes of PWS include paternal deletion (del) of the 15q11.2-13 region, which occurs in approximately 65% of individuals; maternal uniparental disomy (UPD), which occurs in ~30%; and less commonly, an imprinting center defect diagnosed in 3–5% of cases [2]. PWS affects multiple systems, with clinical features that include hypotonia, growth hormone deficiency, hypogonadotropic hypogonadism, sleep disturbances, reduced pain sensitivity, decreased gastrointestinal motility, and scoliosis. A defining feature of PWS, which is rare among neurodevelopmental disorders, is the significant change in eating behavior over time. Whereas infants with PWS have difficulty feeding and may exhibit failure to thrive, a persistent and unregulated desire to eat (hyperphagia) develops during childhood. Unless access to food is strictly controlled, an overwhelming drive to eat coupled with decreased energy expenditure in individuals with PWS leads to morbid obesity. The physiological basis of hyperphagia in PWS remains unclear, and currently, there are no approved medications that effectively treat hyperphagia or other symptoms commonly seen in the syndrome. However, clinical trials testing the effectiveness of novel agents against hyperphagia and related PWS symptoms are currently underway or recently completed [3–11].

In addition to hyperphagia, other behavioral challenges also prominent in PWS include temper outbursts, anxiety, obsessive–compulsive behaviors, rigidity, and social skills (Table 1) [12–18]. These behaviors may or may not be food related. In addition, as individuals with PWS reach adulthood, they are also at high risk for developing psychiatric illness, including psychosis, anxiety disorders, and major depression [19]. The PWS neuropsychiatric phenotype is further complicated by impaired cognition, including intellectual disability, academic difficulties, and specific deficits in social cognition such as perspective taking and reading social/emotional cues [20–22]. Although some clinical trials and intervention studies are occurring, there are distinct challenges to conducting studies in PWS due to the complex interrelated behavioral features. One limitation for the field is the lack of consensus on how to best define or measure many of the complex behaviors associated with PWS. PWS-CTC “Behavior Outcomes Working Group” includes PWS scientists, clinicians, and patient advocacy organization representatives with expertise in the behavioral aspects of PWS. This group sought to address the lack of consensus definitions and descriptions of PWS-associated behavioral features. Using literature review, clinical patient experience, and parent focus groups, the Working Group developed a unified understanding of the key behavioral features in PWS which is presented here. Developing a consensus understanding of the behavioral manifestations in PWS provides an important foundation upon which treatment studies targeting those behaviors can be developed and pursued.

**Table 1:** PWS behavioral features definitions

**Hyperphagia**: intense persistent sensation of hunger accompanied by food preoccupations, an extreme drive to consume food, food-related behavior problems, and a lack of normal satiety	
**Temper outbursts**: highly explosive episodes in which the person with PWS becomes very angry or upset in a way that seems excessive for the situation and also beyond the person’s control	
**Anxiety:** excessive worry and tension often related to schedules/routines, food planning or food security, persons/items of special interest and excessive concerns about the possibility of change	
**Obsessive compulsive behaviors:** repetitive, ritualistic behaviors, collecting and hoarding items, insistence on “sameness,” need to know, ask, or tell	
**Rigidity**: ardent inflexibility with certain routines, concepts, or ways of thinking; vigorous resistance to change; black and white thinking	
**Social cognition:** difficulties relating to others, challenges with reciprocal social communication, recognizing others’ emotions, empathy and accurate interpretation of social cues.	

## Hyperphagia in PWS

### Definition

Hyperphagia in PWS manifests as an intense persistent sensation of hunger accompanied by food preoccupations, an extreme drive to consume food, food-related behavior problems, and a lack of normal satiety. Hyperphagia falls at the far end of a continuum of overeating behaviors and is considered the most extreme form of overeating [23].

### Description

A complex progression characterizes the development of hyperphagia, a hallmark feature of PWS [24]. Infants with PWS present with hypotonia, excessive sleepiness, decreased activity, poor suck, and may show little interest in food. This stage is typically followed by a period of relatively normal growth and weight gain. Subsequently, young children with PWS begin to gain weight easily and show an increasingly strong interest in food. Most individuals go on to develop hyperphagia marked by a lack of normal satiety and an overwhelming drive to eat. The onset of hyperphagia is variable and can occur in early childhood, but the average age of onset is 8 years old [24, 25]. This strong drive to consume food is likely underpinned by persistent feelings of hunger with satiation only developing after excessive calorie intake. Even then, satiety does not persist for any length of time compared to a control group without PWS [26]. Individuals with PWS can readily consume up to three times the normal caloric intake at a given meal, may hoard food, and may eat discarded food [26, 27]. The lack of normal satiety is notable, as exemplified by reported cases of gastric necrosis or stomach rupture arising from extreme overeating [28, 29]. Hyperphagia is life-long in most individuals with PWS, but eating and food interest can wax and wane over time, especially in response to changes in the environment, such as changes in food security or alterations of the food schedule. In general, no consistent differences in hyperphagia have been found between gender or PWS genetic subtypes [30]. However, one small study found that food-related behaviors were more severe in PWS males with deletion [31]. Overall, the severity of hyperphagia is not necessarily correlated with degree of obesity in PWS; access to food is typically the biggest determinant of weight.

PWS-associated hyperphagia has overlap with several other conditions, including binge eating disorder, addiction, and obsessive-compulsive features (preoccupation with food) [32, 33]. However, it is distinguished from these conditions by its presence in early childhood as well as physiological studies suggesting that the hyperphagia in PWS likely is related to a pathological defect of satiety [26, 34–36]. Additionally, a recent study indicated that individuals with PWS display abnormal patterns of neural activity in response to food representations [37].

For families and caregivers, PWS-related hyperphagia is incessant in that once hyperphagia starts, individuals with PWS are constantly in search of food. Thus, for the safety of the person with PWS, restricting access to food and controlling food intake “food security” are paramount and a 24/7 concern for families. Food security may include restricting access to food with locks on refrigerators and pantries, keeping food out of sight and constant supervision, i.e., “eyes on” the person with PWS at all times to ensure they are not seeking/eating food they should not be consuming. This food security must be implemented across all settings, i.e., home, school, work, and social; families must educate all who interact with the person with PWS. Food security also includes schedules and plans for all meals and food consumption. Changes to those plans can lead to significant behavioral challenges for the person with PWS. Families, caregivers, and clinicians have described hyperphagia in PWS as an overwhelming, life-threatening force, which sentences people with PWS to a lifetime of environmental control and restricted lifestyle [23, 38]. Treating hyperphagia is the highest priority for caregivers [39].

### Observable behaviors

Hyperphagic behaviors in PWS include intense focus on food, which can manifest as excessive talking/asking about food, especially details regarding what and when they will eat, and persistently searching for food. The drive to eat can lead to food sneaking/food theft, eating food left on other people’s plates, eating food that is normally considered unacceptable (e.g., food scraps, food in the trash, raw food) or non-food items (e.g., dirt, grass, soap), getting up at night to look for food, taking very large bites of food, and eating very fast [40]. Additional behaviors include becoming agitated or anxious in the presence of food, bargaining for more food, trying to deceive others or lying to obtain food, becoming upset or exhibiting temper outbursts when food is denied, and becoming aggressive to obtain food.

### Assessment of the behavior

The assessment of hyperphagia in the typical population has employed food diaries, visual scales, and test meals in research settings [41]. These methods are considered not useful in PWS due to the individual with PWS’s reluctance to report on their food intake related to concerns about “getting in trouble” and worries about dietary repercussions [42, 43]. Self-report of degree of hunger in individuals with PWS may also be limited by cognitive and communication challenges [44]. In addition, many people with PWS are on calorie-restricted diets; thus, food diaries would not accurately reflect their hunger or hyperphagia. Finally, there are ethical problems of test meals [23] in PWS. And given the physiological impairments in satiety mechanisms in PWS, individuals may not fully experience the contrast between hunger and satiety. Thus, PWS patient-reported hunger scales are considered to be unreliable in this population.

Given the challenges of self-reporting in this population, the field has focused on the use of observer (i.e., caregiver) reported assessment of hyperphagia in PWS. The most frequently used measure of hyperphagia in PWS is the Hyperphagia Questionnaire for Clinical Trials (HQ-CT) [45]. The HQ-CT is a 9-item scale that assesses the severity of specific hyperphagic behaviors including food seeking and food-related preoccupations by assessing distress and adaptive impairment related to hyperphagia. This instrument is modified from a questionnaire developed specifically to quantify hyperphagia in PWS [42], and adapted for use in clinical trials [45]. This observer-reported outcome measure, which aims to measure more objective food-related behavior, is based on behaviors over the past 2 weeks reported by a caregiver who is familiar with the daily activities of the person with PWS. Beyond self- or observer-reported assessment of hyperphagia in PWS, there is interest in the field for developing assessment tools that employ objective, direct measures of food interest, such as eye gaze or evoked response potentials [46, 47].

## Temper outbursts in PWS

### Definition

Temper outbursts in PWS are defined as highly emotional or explosive episodes in which the person with PWS becomes “very angry or upset in a way that seems excessive for the situation and also beyond the person’s control” [48].

### Description

Temper outbursts, sometimes described as emotional outbursts, “tantrums,” or “meltdowns,” are one of the most common maladaptive behaviors reported by parents of children, adolescents, and adults with PWS [5, 6, 37], with a significant impact on quality of life, in some instances equal to or greater than hyperphagia. A small study of PWS families showed a high percentage of siblings experienced PTSD symptoms, and families reported lower overall quality of life compared with normative samples [49]. Additionally, for the individual with PWS, temper outbursts often result in reduced opportunities to socialize in the community, limit living options, and undermine employment. Approximately 80% of individuals with PWS exhibit severe temper outbursts beginning in childhood and persisting throughout adult life [50]. There may be some decrease in frequency of outbursts in adults compared to children with PWS, but adolescents and adults often have outbursts of longer duration than children with PWS [48]. There are no consistent differences in the characteristics of temper outbursts across genders or PWS genetic subtypes [48].

The sequence of behaviors and emotions within temper outbursts in PWS are similar to those seen in typically developing young children, yet the onset begins slightly later in life, and they continue throughout adulthood [48]. These outbursts may be related to “arrested emotional development” in PWS as well to the confluence of hyperphagia, diminished coping skills, poor emotional control/regulation, and limited executive function skills [51–54]. Triggers for temper outbursts in PWS generally fall into the following categories: blocking of a desired goal, a violation of social expectations, perceived injustice, or difficulty dealing with a change in routine [48, 54, 55]. It has been hypothesized that PWS outbursts might be related to hyperphagia. However, Rice et al. [48] found that most parents of young children with PWS reported temper outbursts occurring before the onset of hyperphagia, suggesting outbursts may be developing independent of hyperphagia. One possible mechanism driving the temper outbursts triggered by changes in routine may be a deficit in task-switching ability, a cognitive-related executive function that is an area of particular difficulty in individuals with PWS [56, 57]. Dysregulation of the autonomic nervous system may be another key mechanism underlying temper outbursts in PWS. A small pilot study [58] indicated that stimulation of the vagus nerve decreased temper outbursts for some individuals with PWS, suggesting the role of the autonomic nervous system in this behavioral feature.

### Observable behaviors

Temper outbursts often manifest with the following behaviors: repetitive questioning that may escalate in volume, angry facial expression, increased salivation, talking very loudly, crying, yelling, stomping the feet, slamming doors, kicking, hitting, laying on the ground, and throwing objects/destroying things. Temper outbursts often follow a particular sequence of behaviors starting with emotional behaviors, such as crying and emotional vocalizations. This is rapidly followed by rising anger and more overt behaviors such as verbal and sometimes physical aggression. Early on, it may be possible to distract the individual and avert the full outburst, but there is a point at which the person is unable to bring themselves under control. Temper outbursts may last from minutes to hours before resolving, and there is often a distinct period of “tuning out” or shut down after an outburst and a need for sleep. The sequence may often end with expressions of regret and distress [54].

### Assessment of the behavior

Currently, there are no measures that are validated specifically for the assessment of temper outbursts in PWS; however, several measures have been developed to assess severe behavior challenges in individuals with intellectual disability, which may be applicable to assessing temper outbursts in PWS studies. Among these, the Developmental Behavior Checklist–Monitoring Version (DBC-M) is a caregiver-reported daily diary using items from the Developmental Behavior Checklist [59]. It has been used in a previous clinical trial examining the potential utility of oxytocin for treating behavioral challenges in PWS [53]. Alternatively, the Challenging Behavior Interview has shown utility in a small study assessing the impact of vagus nerve stimulation on temper outbursts in PWS [58]. Finally, the Aberrant Behavior Checklist (ABC-2 [60]; irritability scale) which has been used widely in studies on those with intellectual disability may provide a useful validated measure of temper outbursts in PWS. Bespoke informant diary measures [61] and structured interviews [54] have also been used with some preliminary evidence of validity in PWS.

## Anxiety symptoms and behaviors in PWS

### Definition

Anxiety is common in PWS and is characterized by excessive worry, and tension often related, but not limited to, schedules/routines, food planning or food security, or persons or items of special interest (e.g., teachers, caregivers, pets). Concerns about the possibility of change in these areas are often a trigger for anxiety in PWS.

Anxiety symptoms in PWS can overlap with Generalized Anxiety Disorder (GAD) but also have unique features (e.g., significant worry about food planning/security and items/people of special interest) that are not fully captured using definitions based on the Diagnostic and Statistical Manual of Mental Disorders (DSM-5) [62].

### Description

Parents and clinicians have observed that individuals with PWS may exhibit significant anxiety in both food and non-food-related situations as well as during times of transition and change of routine [57, 63]. However, some studies do not appear to capture the extent of anxiety in the PWS population [64, 65], likely due to challenges of defining and measuring anxiety. Other studies have found moderate to high levels of anxiety in PWS samples [18, 39, 40]. Einfeld et al. [40] found that 43% of individuals with PWS assessed with the Developmental Behavior Checklist (DBC) had significant levels of anxiety. A recent study, Feighan et al. [18], found that 37% of participants with PWS were diagnosed with anxiety (29% of adolescents, 43% of adults), and that anxiety was the most common psychiatric diagnosis in adolescents and adults. Another recent publication examining caregiver preferences for PWS treatment revealed that caregivers rated anxiety as one of their top 2 targets for PWS treatment [39], underscoring the negative impact of anxiety in this population. Finally, a 2018 review of data from the PWS Global Patient Registry [66] found that anxiety symptoms are reported by caregivers to be a significant problem for 48% of the sample of individuals with PWS ages 10 and above [67].

The prevalence of anxiety symptoms in PWS appears to be similar between males and females but higher in individuals with the UPD genetic subtype of PWS compared to the deletion subtype (73% vs. 32%) according to reports of those caring for individuals with PWS [67, 68]. Studies assessing age of onset of anxiety in PWS have not been conducted to date; however, clinical observations suggest that many individuals with PWS struggle with anxiety starting around preschool to school age (3–6 years) with peak anxiety symptoms generally occurring during adolescence/early adulthood [66, 69].

### Observable behaviors

The Diagnostic Manual–Intellectual Disablity-2 (DM-ID-2) [70] speaks to the importance of highlighting the behavioral equivalents of anxiety in the assessment of individuals with intellectual disability and limiting reliance on cognitive and more subjective criteria. Although overt behaviors of anxiety may vary in intensity among individuals with PWS, there is a constellation of explicit behaviors that appear to represent anxiety symptoms commonly expressed in individuals with PWS. These include repetitive questioning especially related to schedules, food and people, pacing, loud and/or fast talking, excessive body and hand movements, trembling, and checking behaviors (e.g., checking on people of particular interest and schedules). In addition, individuals with PWS who are more verbal will frequently express many different worries and feelings of being “stressed” or overwhelmed. It should be noted that there is overlap between anxiety behaviors and compulsive behaviors in PWS as described in the section below.

### Assessment of the behavior

There is no validated standard for measuring anxiety in PWS. Previous research has reviewed tools for measuring anxiety in individuals with intellectual disability via self-report and observer-reported methods [71]. Although some of these measures could be useful in describing anxiety symptoms in PWS, for example, Glasgow Anxiety Scale (GAS-ID) [72] and Anxiety, Depression and Mood Scale (ADAMS) [73], available measures do not appear to fully capture the unique and important manifestations of anxiety in PWS, such as excessive worry about food planning/food security and items/people of special interest. Additionally, given challenges with insight and cognitive difficulties in PWS (e.g., concrete thinking, short term/working memory difficulties), it can be difficult to accurately assess anxiety in this population especially using self-report assessment tools. As noted in the DM-ID2, anxiety symptoms in those with intellectual disability can go undetected by others, and individuals with intellectual disability often do not report symptoms of anxiety [70]. Given these reporting difficulties, caregiver/observer-reported assessments of anxiety are recommended. Previous research and clinical trials have employed the (C)YBOCS [4] to assess anxiety in PWS. Although useful, this measure does not appear to fully capture the range of anxiety symptoms in PWS as it focuses almost exclusively on obsessive thoughts and compulsive behaviors. In response to the lack of effective tools to measure the unique anxiety symptoms and behaviors in PWS, a new measure specific to assessing anxiety and distress in PWS is currently being piloted. The Prader-Willi Syndrome Anxiety and Distress Questionnaire (PADQ) is a 20-item caregiver-rated measure that assesses common indices of anxiety and distress in PWS. The PADQ is currently being used as a secondary outcome measure in phase 3 clinical trial in PWS.

## Obsessive compulsive behaviors in PWS

### Definition

Obsessive-compulsive behaviors (OCB) in PWS are defined as repetitive, ritualistic behaviors that include collecting, storing/hoarding items, insistence on routines, and “sameness”. Other OCB that are common in PWS are “needing to know, ask or tell” about certain information and distress when this behavior is prevented [12, 13, 74–77].

### Description

Anxiety in PWS is often discussed within the context of obsessive compulsive behaviors. Previous studies have found relatively high rates of OC-type symptoms in individuals with PWS with 37–58% having symptoms such as those described above [13]. In a small study, State et al. [76] also found high rates of OC symptoms and concluded that OC symptoms are prominent in the PWS behavioral phenotype. However, in a longitudinal follow-up of over 250 people with PWS, Dykens and Roof [69] found that only 8% of their sample met full DSM-5 criteria [62] for diagnosis of obsessive compulsive disorder (OCD). Many of the repetitive rituals participants with PWS engaged in did not meet the standards for performing behaviors related to obsessive thoughts and fears (illness, germs, cleanliness). Ho and Dimitropoulos [78] also found that the obsessions in PWS often did not map onto the typical preoccupations in OCD such as worries about germs, contamination, religion, or others being harmed. One of the most predominant OC behaviors in PWS is repetitive questioning, which may be related to resistance to change. For families, this behavior can be a source of considerable day-to-day frustration, requiring active behavioral management strategies [79]. Clinical observations and several studies suggest that for many individuals with PWS the OC behaviors may bring enjoyment and are soothing in nature, which is different from what is observed in OCD [80–82]. Additionally, the Diagnostic Manual-Intellectual Disability-2 [70] notes that for many individuals with intellectual disability including PWS, obsessive and compulsive behaviors often do not cause clinically significant distress for the person, although aggressive behaviors may occur if the person is prevented from engaging in the behavior, which is different from classic OCD. Differences between PWS-associated OC behaviors and classic OCD are also supported by neuroimaging studies, which found some similarities but also differences in cerebral circuitry activation in PWS compared to classic OCD [83]. Another principal difference is that in DSM-5 OCD definition, the symptoms are “ego-dystonic,” that is the person has insight that their thinking is irrational but cannot stop the thought or behavior. In most cases of OC behaviors in individuals with PWS, this insight is not present.

There is limited data on age of onset, frequency, or possible genetic subtype differences in OC symptoms in PWS. Clinical observations and parent reports suggest that many individuals with PWS struggle with OC symptoms starting around age 3 [15]. Higher rates of compulsive behavior have also been reported among 2–3 year olds with PWS compared to typically developing children and young children with Down syndrome [15]. The research on differences in OC symptoms among PWS genetic subtypes is inconclusive, with one study finding that overall OC behaviors were higher in the PWS del subgroup, whereas individuals with PWS UPD tended to have higher rates of OC behaviors that mimic those commonly observed in autism spectrum disorder (ASD) [84].

### Observable behaviors

Obsessive compulsive behaviors in PWS vary in intensity and scope. However, as described above, there is a constellation of behaviors that are common in this population, including insistence on “sameness” in certain routines and need to know/tell/ask, collecting items of interest/“hoarding,” checking behaviors (e.g., checking on collected items of interest), and repetitive rewriting, arranging, and rearranging.

### Assessment of the behavior

Previous research and clinical trials in PWS have employed the Children’s Yale Brown Obsessive Compulsive Scale [4, 85] to assess OC behaviors. The CY-BOCS is a well-validated frequently used measure of OCD but includes some items that are not pertinent to PWS. Although helpful in describing some of the salient OC symptoms in PWS, the CY-BOCS does not capture the broader manifestations of anxiety in PWS as noted above.

## Rigidity in PWS

### Definition

Rigidity in PWS is defined as ardent inflexibility with respect to certain routines, concepts, or ways of thinking with vigorous resistance to change. Rigidity can also be characterized as “black & white thinking” and a resistance to considering information or evidence that might conflict with the rigidly held belief. People with PWS can demonstrate both cognitive and behavioral rigidity.

### Description

Parents and clinical experts report that people with PWS often have rigid thinking styles and behaviors that interfere with daily functioning. Individuals with PWS commonly show a strong resistance to change [17]. This difficulty with change of routine and inflexibility in PWS may be linked to a cognitive deficit in task switching [56, 57]. Task switching requires being able to move from one task to a different task in a timely manner, an area of difficulty for individuals with PWS [56, 86].

Studies assessing age of onset of rigidity behaviors in PWS have not been conducted to date. However, clinical observations suggest that many individuals with PWS exhibit behaviors reflecting resistance to change and inflexibility at a young age, generally before starting school. Dykens et al. [87] found that compulsivity and insistence on sameness in routines or events were seen in 76–100% of the participants with PWS (4–21 years). Little is known about the variation of these behaviors over time, gender, or genetic subtype differences. It is notable that although individuals with PWS have a range of intellectual functioning from moderate intellectual disability (ID) to normal IQ (with some learning challenges), the symptoms of rigidity in PWS are seen across the spectrum of intellectual function in PWS, not just in those with lower (or higher) IQ levels. Clinically, these behaviors have a significant impact on adaptive functioning and are among some of the most difficult behaviors for parents to manage.

### Observable behaviors

Overt behaviors that appear to reflect behavioral rigidity in PWS can include needing to have things done a certain way, including in a particular order, time, or place; difficulty with changes in the schedule or routine; and difficulty transitioning from one activity to another (e.g., brother must always sit in the seat behind the driver, hair must be worn a certain way every day, refusal to take medication if not given the right way). Rigidity can be observed by the person with PWS getting stuck on a certain thought or idea and excessive talking about that topic. Additional characteristics include inflexibility in beliefs and difficulty considering a new perspective or topic even when presented with conflicting information (e.g., only females can be teachers but their teacher is male). Another common observable behavior reflecting rigidity in PWS is significant distress and protest when the rigid behaviors, thoughts, or beliefs are challenged. Additionally, protest and distress behaviors in PWS are often seen when the person with PWS expects something that then does not happen.

### Assessment of the behavior

Commonly used behavior scales (e.g., CY-BOCS, Repetitive Behavior Scale-Revised- RBS-R) do not specifically focus on rigidity. Two domains of the RBS-R do assess needs for sameness and compulsivity in those with autism spectrum disorder (ASD) or ID yet are not specifically tailored to PWS. Some of the rigid behaviors seen in PWS could be captured on the CY-BOCS, but the instrument does not adequately capture the majority of these behaviors and does not focus on cognitive rigidity.

The Montefiore-Einstein Rigidity Scale (MERS-PWS) is currently being developed to capture the above described rigidity characteristic of PWS. The MERS-PWS scale measures 3 domains that reflect rigidity: behavioral rigidity, cognitive rigidity, and a protest domain. The MERS-PWS is currently being tested as a secondary outcome measure in a clinical trial examining challenging behaviors in adolescents and young adults with PWS.

## Social cognition deficits in PWS

### Definition

Individuals with PWS display significant difficulties relating to others, especially peers. The social difficulties seen in PWS are characterized by challenges with reciprocal social communication, recognizing others’ emotions, empathy, and accurate interpretation of social cues. Social skills challenges in PWS can overlap with those observed in ASD and are influenced by genetic subtype.

### Description

Individuals with PWS struggle with reciprocal social communication and with recognizing emotions in others, especially negative emotions such as sadness, anger, and fear [88]. Related to this, individuals with PWS have difficulty accurately processing faces [21, 89], interpreting emotional valence in faces, being aware of other’s personal space, and show significant developmental delay in “theory of mind” tasks [50, 89–91]. In a recent study, Dykens et al. [88] found that individuals with PWS also have difficulties with accurately perceiving the intentions of others. The above social difficulties could be due to differences in the way individuals with PWS process incoming information, which could impact on social skills. Interestingly, a physiological basis for these deficits is likely, with genetic subtype differences evident in several studies. Individuals with the uniparental disomy [UPD] or imprinting defect [ID] genetic subtypes tend to have more difficulty with social communication and are more likely to be diagnosed with ASD; however, all genetic subtypes of PWS (deletion, UPD, ID) show social challenges [20, 89]. To date, no studies have found significant gender differences in social skills challenges in PWS.

Although there is overlap between the social difficulties seen in PWS and those observed in ASD, the prevalence and nature of ASD in PWS has been the subject of some debate among clinicians and researchers. Estimates of ASD in people with PWS has been as high as 41% [92]; however, Dykens et al. [87] used multimodal forms of assessment including direct assessments of the person with PWS and an expert clinical panel and found a rate of ASD diagnosis of just 12.3%. The majority of those meeting ASD criteria having the UPD genetic subtype. The authors suggested that high rates of ASD in previous studies were based solely on parent-reported screening questionnaires and thus may have overestimated rates of ASD diagnosis. It should be noted, however, that although most of the children with PWS assessed in the Dykens et al. [87] study did not meet criteria for ASD, a large majority had some level of social skill difficulties, especially in the area of reciprocal social interactions.

### Observable behaviors

Observable behaviors that reflect the social skills challenges in PWS can manifest as lack of or minimal reciprocal conversation. The person with PWS typically focuses the conversation on a narrow range of topics they are interested in, with repetitive features in the conversation. People with PWS may tend to play with younger children and struggle more with peers. Difficulty in maintaining personal space (e.g., standing too close) is also common [89]. Social cognitive difficulties have been shown in children as young as 3 years old [93]. Older children and adults with PWS may behave in ways that reflect feelings of being easily slighted; they may be quick to become frustrated or angry and take things personally. People with PWS often have difficulty modifying their social behavior to fit changing situations as they arise. Temper outbursts, difficulties with routine change, and ritualistic and repetitive behaviors, as mentioned above, are common in PWS and present unique challenges in the social arena.

### Assessment of the behaviors

Currently, there are no measures that are specific to assess social skills challenges in PWS. However, some studies exploring social challenges in PWS have employed the Social Responsiveness Scale (SRS) and the Social Competence Inventory [94], which are parent-rated inventories [89]. These are well-validated measures to assess social challenges in those with ASD or ID. Other studies have used emotion recognition tasks or vignettes to assess social perceptions. Some of the social challenges seen in PWS are captured by these measures, but they do not adequately assess the breadth of unique social challenges seen in PWS. A combination of parent, clinician, and teacher rating scales, in addition to direct patient measures (e.g., face/emotion tasks), may be necessary to fully capture the nature of social skills challenges in PWS. Tools like the ADOS-2 [95] and other clinician-rated assessments are recommended especially when assessing for ASD. Relying on parent assessments only may give an incomplete picture of the social skills deficits observed in PWS [87].

### Overlap of PWS behavioral symptoms and features

**Fig. 1:**
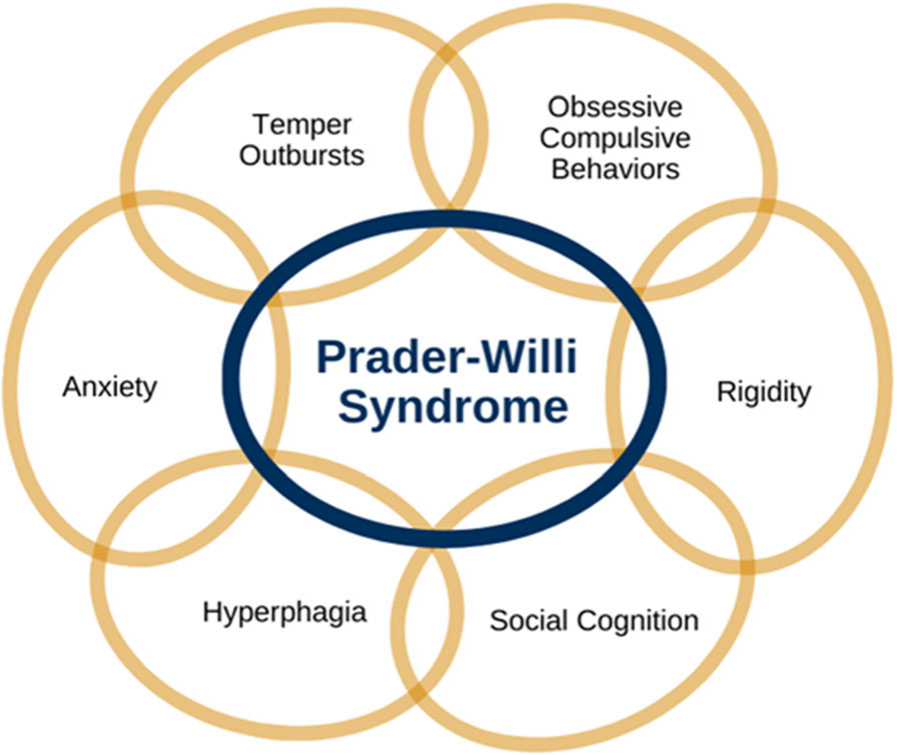
Key interrelated behavioral features of PWS

As is apparent from the above descriptions, there can be significant overlap of features among the various behavioral characteristics in PWS (Fig. 1). It may not be feasible to tease apart these features as many individuals with PWS struggle with multiple behavioral challenges simultaneously. As an example, the anxiety, OC behaviors, rigidity, and temper outbursts observed in PWS appear to have significant overlap. One theory is that similar psychological or physiological phenomenon and/or cognitive mechanisms, such as difficulties with task switching or emotion regulation, may underlie these behavioral challenges. However, further research is needed to advance our understanding of the possible mechanisms underlying these key behavioral features in PWS. Overlap of symptoms in mental health and behavioral disorders can be seen in the general population as well, for example, in anxiety and depressive mood disorders [96]. This overlap of symptoms and behaviors across different mental health conditions and features may occur due to universal dimensions of distress or negative affect, as well as to shared genetic and neurobiological aspects of these mental health conditions. This is likely a factor in PWS as well. However, in PWS, it may not be possible to separate out aspects of each of the behavioral challenges unless and until we have effective treatments for that feature. Additionally, one treatment in PWS may address multiple behavioral features seen in the syndrome related to overlapping neurobiological mechanisms and/or genetic factors.

## Conclusions and limitations

The primary aim of this paper is to review the current knowledge base regarding the defining features of the behavioral phenotype in PWS and provide consensus definitions of these behavioral features based on the work of the PWS-CTC Behavior Outcomes Working Group. Developing a unified understanding of the PWS behavioral phenotype is important as interventions and treatments for PWS are moving forward, and the behaviors reviewed in this paper are currently or likely soon to be targets of treatment studies. This paper also highlighted some of the assessment tools that are currently available and under development, which may be useful in measuring these features in PWS. Although the current paper discusses the use of several measurement tools, it was beyond the scope of this paper to provide a complete assessment of available behavioral assessment tools and their potential utility in PWS. This paper represents only an initial foray into this area. Future studies are needed to determine if existing measures for the assessment of behavioral challenges in individuals with intellectual disability can be validated for use in PWS or whether new PWS-specific assessments are needed. Further work on the utility of advancing more appropriate measures for PWS will be an ongoing focus for the PWS-CTC as well as for the field as a whole.

## PWS patient vignettes

The following are PWS patient vignettes to highlight the behavioral features discussed in this paper. These vignettes are not based on any one patient with PWS but rather a combination of different behaviors observed by clinicians with experience working with individuals with PWS.

### Feature: hyperphagia

1. B. is an 18-year-old male with PWS living at home with parents and sister. He admits he is hungry “all the time” but would not admit to taking food around the house or at school. He has taken bolt cutters from the garage to break the locks on the pantry and has also used a screwdriver to take the hinges off locked cabinets to try to get at food. He has convinced classmates that he did not have a lunch after he ate it on the morning bus ride to school. He talks and thinks about food constantly but denies eating food when his mom finds wrappers stuffed in his sneakers. He is clever and fast when it comes to obtaining food; he once stole more than 200 dollars from classmates during a play rehearsal to buy food from a convenience store. Because he is so friendly and social, none of the kids thought he could have done it. They often give him snacks and other food, because he asks “so nicely” even though they know he should not have it.
2. J. is a 10-year-old girl with PWS who was at her brother’s baseball game, sitting in the stands with her mother. When her doll fell under the bleachers, her mother let her go down and get it since she felt she could have “eyes on her” the entire time. As she returned from getting her doll, she reached into a backpack that was near the coaching bench and quickly took something out and poured it in her mouth, swallowing hard. Her mother ran to her and asked her what she ate but she put her hands over her mouth and would not talk. Her mother raised her voice in tears asking, “What did you get?” Everyone at the ballpark was looking at them. J never admitted to what she ate but had food around her mouth and hands.

### Feature: temper outbursts

1. K is a 22-year-old male with PWS living at home with parents and sister. He has aged out of school services and is now waiting for vocational services. He began having temper tantrums at age 8, and they have gotten progressively worse, with broken furniture, scared family members, and calls to police. For example, K became upset when a sporting event was rained out and he had to leave the field unexpectedly. He became more agitated on the drive home, cursing and asking “why” he had to go home. His parents’ explanations about lightning and possible injury or death could not dissuade him from the “unfairness” of the situation. He came home, went to his room and within minutes had broken a lamp, his glasses, and ripped his shirt. He came out and threatened his mom and tried to hit her with a book. Dad intervened and finally had to sit on his 150-lb son to keep him from injuring himself or others. Police have been called by neighbors and his own parents, but besides taking him to the station or patrol car for a “scared straight moment,” they have no answers. He is usually quite tearful and remorseful after these tantrums blow over but admits he cannot control them when they are happening.
2. J is a 10-year-old girl living with PWS. While at school 1 day shortly after recess started, one of the teachers noticed J laying on the ground yelling, swatting, and biting anyone close to her. Then, a loud pitched scream came from her that stopped the playground. J then got up and started running, knocking anyone and anything in her path. She then curled up in a ball on the other side of the field and the teachers told everyone to give J space. She finally calmed and then sat picking handfuls of grass. The vice principal came out to talk to J who at first would not speak. As the principal talked quietly to her, J. calmed down but seemed not to recall the event. When asked how she was feeling, she said, “Fine.” She then got up to walk back to her classroom. The principal noted that these episodes happen frequently.

### Feature: obsessive compulsive behaviors

A is a 16-year-old girl with PWS who lives at home with her parents, attends a local high school, and receives special education services. Her father reports that A collects and hoards hundreds of rubber gloves and spends countless hours sorting, piling, and rolling them into tight balls and then puts them in bins. She does not use the gloves. She does this activity for hours after school, and if her father walks into her room, she must check the sole of his shoes and pockets to make sure that none of her gloves have been inadvertently “picked up”. If her parents try to limit this activity or keep her from obtaining more gloves, she becomes hysterical and sometimes aggressive. She even insists on taking them on family trips and recently to her grandfather’s funeral.

### Feature: anxiety symptoms

M is an 11-year-old girl with PWS living with her parents and three siblings. She spends hours a day confirming the daily schedule, asking many questions about who will carry out daily care and academic activities, and needs to check in constantly with mom and a full-time care assistant. She becomes tearful if her mom reminds her that her schedule is the same every day and the same people will be helping her. She asks, “but what if it’s not?” What if’s rule her life, and M shuts down if caregivers try to discuss this with her. If they persist in this M.’s behaviors escalate to crying and yelling. M asks more than 300 questions a day and does not trust her teacher, aides, and other school staff to help. The school had to call mom every day several times/day because of this. Her anxiety behaviors at school became unmanageable and her mom has chosen to homeschool her now. M is on more than 3 psychiatric medications, which have helped “take the edge off,” but have not reduced her anxiety to allow for increased functioning for M.

### Feature: rigidity

P is a 23-year-old male living with his parents. He went to a local school and graduated with a regular diploma, fulfilling all state requirements, and he yearns for a “real job”. He has been unable to keep employment because of his rigid behaviors. P feels that tasks should be completed a certain way and is taken to arguing with coworkers and bosses when he feels they are “wrong”. He once was fired after arguing with a customer about which dog food is best. P tried unsuccessfully to convince the customer that the food they wanted was not as good for their dog as another brand. The customer tried to be good-natured, but eventually asked for a manager. P then tried to convince the manager “that no one knows more about dogs than me”. He has many strongly held beliefs on everything (from best pizza in Baltimore to worst gorilla exhibit in the USA) and spends a great deal of time trying to convince others that he is right. These beliefs often are based on things he has read and facts that he believes. The preferences and opinions of others do not deter him of his mission that he is right and that others are wrong. His parents are worried that he won’t be able to ever maintain employment or social connections because he cannot be dissuaded and that he cannot avoid arguing with and offending others.

## Data Availability

Not applicable

## Abbreviations

PWS: Prader-Willi syndrome
UPD: Uniparental disomy genetic subtype
Del: Deletion genetic subtype
OCD: Obsessive compulsive disorder
OCB: Obsessive compulsive behaviors
ASD: Autism spectrum disorder
ID: Intellectual disability
